# Disposable Polyaniline/*m*-Phenylenediamine-Based Electrochemical Lactate Biosensor for Early Sepsis Diagnosis

**DOI:** 10.3390/polym16040473

**Published:** 2024-02-08

**Authors:** Piromya Thongkhao, Apon Numnuam, Pasarat Khongkow, Surasak Sangkhathat, Tonghathai Phairatana

**Affiliations:** 1 Department of Biomedical Sciences and Biomedical Engineering, Faculty of Medicine, Prince of Songkla University, Hat Yai, Songkhla 90110, Thailand; 2 Center of Excellence for Trace Analysis and Biosensor, Prince of Songkla University, Songkhla 90110, Thailand; 3 Division of Physical Science, Faculty of Science, Prince of Songkla University, Songkhla 90110, Thailand; 4 Translational Medicine Research Center, Faculty of Medicine, Prince of Songkla University, Songkhla 90110, Thailand; 5 Institute of Biomedical Engineering, Faculty of Medicine, Prince of Songkla University, Hat Yai, Songkhla 90110, Thailand; 6 Department of Surgery, Faculty of Medicine, Prince of Songkla University, Songkhla 90110, Thailand

**Keywords:** biosensor, electrochemistry, lactate, layer-by-layer, *m*-phenylenediamine, polyaniline, point-of-care testing, sepsis

## Abstract

Lactate serves as a crucial biomarker that indicates sepsis assessment in critically ill patients. A rapid, accurate, and portable analytical device for lactate detection is required. This work developed a stepwise polyurethane–polyaniline–*m*-phenylenediamine via a layer-by-layer based electrochemical biosensor, using a screen-printed gold electrode for lactate determination in blood samples. The developed lactate biosensor was electrochemically fabricated with layers of *m*-phenylenediamine, polyaniline, a crosslinking of a small amount of lactate oxidase via glutaraldehyde, and polyurethane as an outer membrane. The lactate determination using amperometry revealed the biosensor’s performance with a wide linear range of 0.20–5.0 mmol L^−1^, a sensitivity of 12.17 ± 0.02 µA·mmol^−1^·L·cm^−2^, and a detection limit of 7.9 µmol L^−1^. The developed biosensor exhibited a fast response time of 5 s, high selectivity, excellent long-term storage stability over 10 weeks, and good reproducibility with 3.74% RSD. Additionally, the determination of lactate in human blood plasma using the developed lactate biosensor was examined. The results were in agreement with the enzymatic colorimetric gold standard method (*p* > 0.05). Our developed biosensor provides efficiency, reliability, and is a great potential tool for advancing lactate point-of-care testing applications in the early diagnosis of sepsis.

## 1. Introduction

Sepsis is a time-critical medical and life-threatening condition that occurs as the body’s response to an infection. It can further progress to sepsis shock, eventually leading to severe organ dysfunction and death in the emergency department [1,2]. Over 48.9 million people globally are undergoing sepsis and one-fifth of these patients have not survived [3]. Sepsis patients also have non-specific symptoms and poor prognostic pathology. According to the Surviving Sepsis Campaign, blood lactate level or blood lactate clearance is one of the most important biomarkers used for the evaluation of critically ill patients [4]. An increase in the blood lactate level is related to the high morbidity and mortality rate among ill patients. A resting blood lactate level is normally accumulated in the range of 0.5–2.2 mmol L^−1^ [5]. A lactate level of more than 4 mmol L^−1^ is considered to be a high risk for mortality. The impairment of lactate generation and clearance, particularly in critically ill patients, can cause the accumulation of blood lactate that can be found at >4.0 mmol L^−1^ [6,7]. Thus, early detection of lactate levels can reduce the mortality rate.

Techniques for lactate determination include automated devices based on enzymatic colorimetry and arterial blood gas analysis (ABG) [8]. Although these techniques are used in several clinical laboratories, they are expensive and need complicated procedures that require sample preparation by trained personnel, making them time-consuming [9]. In addition, ABG can also cause a high-risk infection when the blood sample is frequently collected from an artery [10]. To overcome these issues, electrochemical biosensors are being explored due to their high sensitivity, selectivity, simplicity, rapid response, cost-effectiveness, lack of requirement for sample preparation, and the considerable ease in developing a portable device for point-of-care (POC) diagnosis [11,12,13].

There are numerous research groups attempting to develop electrochemical biosensors for lactate analysis in blood samples [14,15]. However, achieving a reproducible detection range that is associated with sepsis remains a challenge. To tackle this problem, most research groups have focused on electrode modification through enzyme immobilization [16]. This approach offers a significant advantage by preserving the biosensor’s stability [17], thereby enabling its application in the analysis of blood samples that demand high selectivity. Additionally, it contributes to achieving a linear range suitable for medical applications, characterized by good reproducibility [15].

An essential strategy for enzyme immobilization involves the careful selection of materials that confer unique properties to enhance sensitivity, stability, and biocompatibility. Polymers stand out as particularly attractive in this context due to their excellent suitability as supporting materials for biorecognition immobilization. Furthermore, they offer the added benefit of allowing precise control of thickness through electropolymerization [18]. Among them, polyaniline (PANI), a conducting polymer, is widely used in biosensor design [19,20]. This is because of its unique properties, i.e., tunable electrochemical characteristics, functionality-rich chemical structure, providing the amino groups (–NH_2_), increasing specific surface area, high conductivity, biocompatibility, and great long-term stability [21,22,23,24]. Similar to PANI, *m*-phenylenediamine (*m*-PD) is another intriguing component in the construction of biosensors and various electrochemical devices. Its contribution of amino groups (–NH_2_) to the molecular structure and enhancement of specific surface area further elevates its significance in the realm of biosensor design. Much research has also used it as a perm-selective membrane to eliminate the interfering species [15,18,25,26].

In this study, we introduced a novel electrochemical lactate oxidase-based biosensor that utilized a layer-by-layer method to modify a screen-printed gold electrode. The layers were made of three distinct polymers, including *m*-PD, PANI, and polyurethane (PU). Specifically, PU was coated as an outer layer to prevent the enzyme from leaching from the sensor, and to ensure that the detection linearity covered the lactate range associated with sepsis. The experiment parameters regarding the layer-by-layer modification were investigated and optimized, i.e., PANI thickness, PU concentration, and applied potential of lactate detection. Finally, the developed lactate biosensor was examined for human plasma analysis and compared to the results obtained from the gold standard-based enzymatic colorimetric method used in hospitals.

## 2. Materials and Methods

### 2.1. Reagents and Apparatus

Lactate oxidase (LOx) from *Aerococcus viridans* (41 units mg^−1^ lyophilized powder) was purchased from Sigma-Aldrich (St. Louis, MO, USA). Aniline (≥99.5%), *m*-Phenylenediamine (*m*-PD, Flakes, 99%), polyurethane (PU, Selectophore^TM^), glutaraldehyde (GA, 25% in H_2_O solution), bovine serum albumin (BSA, pH 7, ≥98%), sodium lactate, uric acid (≥99%, crystalline), D-(+)-glucose, dopamine, L-ascorbic acid (99%), and potassium hexacyanoferrate (III) (K_3_[Fe(CN)_6_]) were from Sigma-Aldrich (USA). Sodium dihydrogen orthophosphate (NaH_2_PO_4_), di-sodium hydrogen orthophosphate (Na_2_HPO_4_), and potassium chloride (KCl) were from Ajax Fine chem (New South Wales, Australia). Hydrochloric acid (HCl, 37%), dimethylformamide (DMF), and tetrahydrofuran (THF) were from Merck (Darmstadt, Germany). Other chemicals were of analytical grade. All aqueous solutions were prepared with water from Milli-Q purification system (resistivity ≥ 18 MΩ cm^−1^).

Electrochemical experiments were performed using an Autolab Potentiostat/Galvanostat controlled by the NOVA 2.1.4 software (Metrohm Autolab, Utrecht, The Netherlands). The measurements were carried out using the screen-printed gold electrode (Metrohm, KM Utrecht, The Netherlands) comprised of a gold working electrode (4 mm in diameter, 0.126 cm^2^), a silver pseudo-reference electrode, and a gold counter electrode [27]. Scanning electron microscopic images were obtained using a scanning electron microscope (SEM, Quanta 400, FEI, Osaka, Japan). All experiments were carried out at room temperature (25 °C).

### 2.2. Lactate Biosensor Fabrication

**Figure 1:**
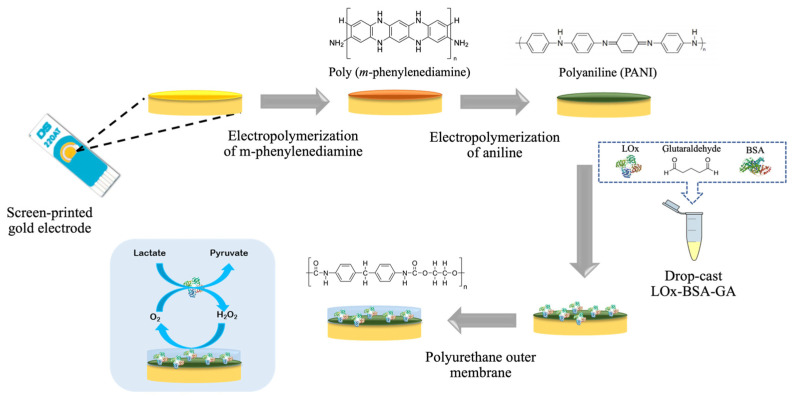
Schematic illustration of the lactate biosensor fabrication using layer-by-layer method.

Our developed lactate biosensor was constructed on a screen-printed gold electrode (SPAuE) using *m*-phenylenediamine (*m*-PD), polyaniline (PANI), and polyurethane (PU) based on layer-by-layer method. Prior to the electrode modification, the SPAuE was electrochemically pre-treated in 0.50 mol L^−1^ H_2_SO_4_ using cyclic voltammetry with potential scanning from 0.0 V to 1.1 V at a scan rate of 100 mV s^−1^ for 10 cycles, and was rinsed with deionized (DI) water. The step of electrode modification is illustrated in Figure 1. Initially, *m*-PD as a perm-selective membrane was deposited onto the prepared SPAuE via electropolymerization. This was accomplished using chronoamperometric method by applying a constant potential of 0.70 V for 20 min in 0.1 mol L^−1^ *m*-PD in 0.01 mol L^−1^ phosphate buffer solution (PBS, pH 7.4), followed by rinsing with DI water (*m*-PD/SPAuE). Next, a PANI layer was prepared on the *m*-PD/SPAuE by electropolymerizing 55 mmol L^−1^ aniline in 1.0 mol L^−1^ HCl, applying the potential range from −0.20 V to 1.0 V at a scan rate of 50 mV s^−1^ for 20 cycles (PANI/*m*-PD/SPAuE). Subsequently, an enzyme mixture comprising 20 µL of LOx (0.3 U), 2 µL of glutaraldehyde (GA, 2.5% *v*/*v*) as a crosslinker, and 5.0 µL of 250 mg mL^−1^ BSA as a protein stabilizer to preserve enzyme activity was prepared. A volume of 4.0 µL of this mixture was drop-casted onto the PANI/*m*-PD/SPAuE. The modified electrode was left at room temperature for an hour and then placed in a sealed system at 4 °C overnight for the crosslinking process (LOx/PANI/*m*-PD/SPAuE). Finally, the LOx/PANI/*m*-PD/SPAuE was coated with 2.0 µL of PU (2.0% *w*/*v* in THF/DMF solution) as an outer layer (PU/LOx/PANI/*m*-PD/SPAuE). The fabricated lactate biosensor was rinsed with PBS before testing and stored in a sealed system containing 0.1 mol L^−1^ PBS at 4 °C when not in use.

### 2.3. Electrochemical Measurement

To verify that the working electrode had been successfully coated, each step of electrode modification was examined in a solution containing 5.0 mmol L^−1^ K_3_Fe(CN)_6_ and 0.10 mol L^−1^ KCl using cyclic voltammetry in the range of −0.20 V to 0.40 V at a scan rate of 50 mV s^−1^. The lactate determination was conducted via amperometry at a constant potential of +0.70 V under stirring conditions. The amperometric response was measured as the increase in current, which was proportional to the concentration of lactate.

### 2.4. Optimization Studies

A set of parameters influencing the developed biosensor was studied and optimized in the range of 0.20 to 1.0 mmol L^−1^ lactate concentrations. These parameters included the strategies of polymer electrode modification, the thickness of PANI, PU concentrations, and the applied potential. Initially, four different strategies of electrode modification based on components and their order were explored, including (1) LOx/*m*-PD/SPAuE, (2) LOx/PANI/SPAuE, (3) LOx/PANI/*m*-PD/SPAuE, and (4) LOx/*m*-PD/PANI/SPAuE. The thickness of PANI was then optimized for the scan number of electropolymerization at 10, 20, 30, and 40 cycles. For PU layer, it was investigated by varying the concentration of PU (%*w*/*v*) at 1.0%, 2.0%, and 3.0%. Additionally, the effect of applying potential for lactate detection at 0.50, 0.60, 0.70, and 0.80 V was also observed. The optimum conditions were considered based on achieving the highest sensitivity (the slope of the calibration plot), a board linear range capable of covering the sepsis range, and a short response time.

### 2.5. Selectivity, Reproducibility, Long-Term and Storage Stability

The selectivity of the developed biosensor to lactate in the presence of potential blood-interfering substances at high physiological concentrations was examined through amperometric detection. These substances included 0.10 mmol L^−1^ ascorbic acid (AA), 0.10 mmol L^−1^ uric acid (UA), 0.10 mmol L^−1^ dopamine (DA), and 5.0 mmol L^−1^ glucose (Glu).

The reproducibility test was assessed using six different electrodes, measuring their amperometric responses to a range of lactate concentrations (0.20–5.0 mmol L^−1^). The long-term stability of the biosensor was tested weekly by comparing the sensitivity of subsequently used electrodes to their initial values. Storage stability was also investigated using nine lactate biosensors prepared simultaneously, with their sensitivities examined through random testing every 3–5 days over a period of two months. Following each testing step, the biosensor was cleaned and stored in a sealed system containing 0.1 mol L^−1^ PBS at 4 °C when not in use.

### 2.6. Real Sample Analysis

Human blood plasma samples received from Songklanagarind Hospital, Hat Yai, Thailand (approved by the local ethics committee [REC.63-161-25-2]) were analyzed. Initially, the matrix effect was studied by adding a series of lactate concentrations at 4.0, 6.0, 10.0, 20.0, and 30.0 mmol L^−1^ into the tubes containing blood plasma samples. Then, the mixture (lactate and blood) was diluted with 0.1 mol L^−1^ PBS (pH 7.4) to obtain a 10-fold dilution (spiked sample). The matrix effect was determined by comparing the slope of the calibration curve (sensitivity vs. concentration of lactate) using the standard lactate solution, with that using the spiked sample through two-way ANOVA. To ascertain the lactate concentration in plasma samples using the developed lactate biosensors, eight blood plasma samples were tested, and the results were compared with values obtained from the gold standard enzymatic colorimetric method using Cobas c502 analyzer (Roche, Basel, Switzerland) used in hospitals. The comparison was conducted using the Wilcoxon signed-rank test.

## 3. Results and Discussion

### 3.1. Characterization of the Lactate Biosensor

#### 3.1.1. Surface Morphology

**Figure 2:**
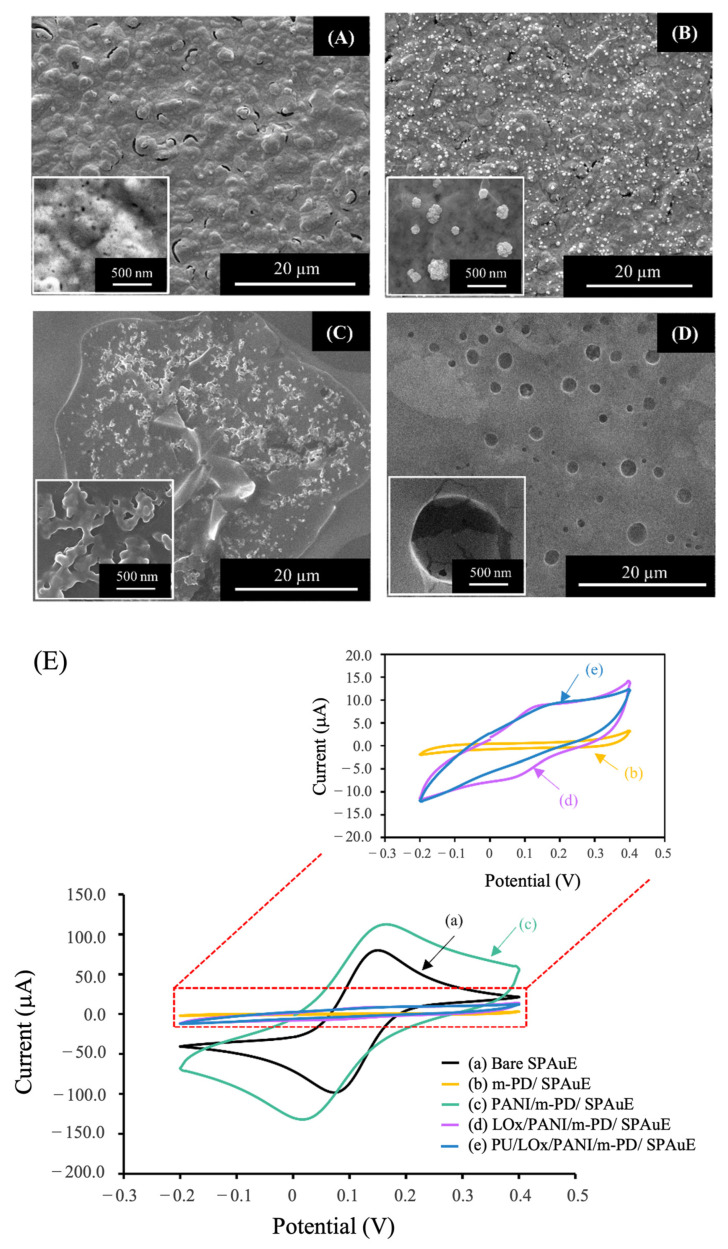
SEM images of the modified SPAuE: (**A**) *m*-PD/SPAuE, (**B**) PANI/*m*-PD/SPAuE, (**C**) LOx/PANI/*m*-PD/SPAuE, and (**D**) PU/LOx/PANI/*m*-PD/SPAuE, (**E**) cyclic voltammograms of the modified electrode in each fabrication step performing in 5.0 mmol L^−1^ [Fe(CN)_6_]^3−/4−^ solution containing 0.10 mol L^−1^ KCl at a scan rate of 50 mV s^−1^.

The surface morphology of the modified electrode in each fabrication step was examined using SEM. Figure 2A shows a rough surface of the *m*-PD structure coated on the electrode surface (*m*-PD/SPAuE). After PANI electropolymerization, the distribution of the PANI granules with an average diameter of 390 ± 18 nm on the surface of the *m*-PD/SPAuE was observed (Figure 2B). Figure 2C presents the rough, gel-like layer of the enzyme matrix (LOx via the crosslinking reagent and BSA) coated on the PANI/*m*-PD/SPAuE. When the PU as an outer layer was coated, a smooth surface with bulky pores was observed (Figure 2D), similar to those reported earlier [28]. These SEM images could indicate that each fabrication step of the electrode modification was achieved.

#### 3.1.2. Electrochemical Characterization

To confirm the successful fabrication of the developed lactate biosensor, the stepwise modification was electrochemically characterized using cyclic voltammetry in a redox solution of 5 mmol L^−1^ K_3_Fe(CN)_6_ at a scan rate of 50 mV s^−1^, as illustrated in Figure 2E. The cyclic voltammogram (CV) of the bare SPAuE (trace a) exhibited a pair of well-defined redox peaks. When the *m*-PD layer was deposited on the electrode surface, the CV became a flat shape without the redox peaks (trace b), indicating the successful coating of the non-conductive *m*-PD layer on the SPAuE. Subsequently, after coating PANI onto the *m*-PD/SPAuE, the highest background current with a couple of redox peaks was observed (curve c). This indicated that PANI provided a conductive layer on the electrode surface which could be attributed to electron transfer onto the electrode surface, confirming the successful coating of PANI on the previous layer. Upon enzyme immobilization through the co-crosslink of glutaraldehyde and BSA (non-conducting materials), a decrease in the redox current signals was observed (curve d). This reduction was attributed to the non-conductive nature of the enzyme matrix, blocking electron transfer toward the electrode surface [29]. Finally, when the non-conductive PU was drop-casted, the current response slightly decreased, indicating the successful coating of the PU layer as an outer membrane onto the LOx/PANI/*m*-PD/SPAuE (curve e).

### 3.2. Optimization Studies

#### 3.2.1. Effect of Electrode Modification Strategies

**Figure 3:**
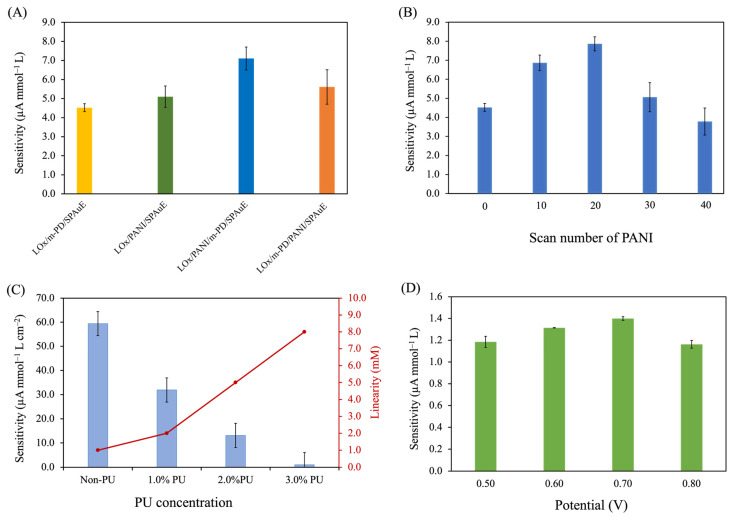
Optimization studies on the sensitivity for different lactate biosensor fabrication (n = 3): effects of (**A**) the components and the sequence of electrode modification, (**B**) scan cycles of PANI electropolymerization, (**C**) concentrations of PU compared with the linearity range of lactate measurements, and (**D**) constant applied potentials for lactate determination.

To investigate the effect of component and the sequence of PANI and *m*-PD on SPAuE, four strategies of electrode modification, i.e., (1) LOx/*m*-PD/SPAuE, (2) LOx/PANI/SPAuE, (3) LOx/PANI/*m*-PD/SPAuE, and (4) LOx/*m*-PD/PANI/SPAuE were examined based on their sensitivity using amperometry at a constant potential of 0.70 V. As seen in Figure 3A, the results revealed the significant impact of both components and the sequence of electrode modification on sensitivity. Among these modifications, the LOx/PANI/*m*-PD/SPAuE exhibited the highest sensitivity for lactate detection, followed by the LOx/*m*-PD/PANI/SPAuE, LOx/PANI/SPAuE, and LOx/*m*-PD/SPAuE, respectively. The use of *m*-PD as a supporting material for LOx immobilization (LOx/*m*-PD/SPAuE) resulted in lower sensitivity compared to PANI without *m*-PD (LOx/PANI/SPAuE). This could be attributed to the non-conductive nature of *m*-PD, coupled with its thin film, potentially affecting the surface area available for LOx immobilization. Moreover, the combination of *m*-PD and PANI in lactate biosensor fabrication was found to enhance sensitivity. PANI, known for its high conductivity, positively influenced current signal response, electrocatalysis, and the kinetics of the electron transfer process to the electrode surface [30]. The chemical structure of PANI, presenting amino group (–NH_2_), offered a large specific surface area in granular form for enzyme immobilization. The amino groups also provided by *m*-PD coating further contributed to this effect. The synergistic effect of both *m*-PD and PANI could contribute to an increase in the specific surface area for a large amount of lactate oxidase loading, resulting in an elevated current response. Consequently, LOx/PANI/*m*-PD/SPAuE was chosen for further experiments.

#### 3.2.2. Effect of the PANI Layer

The PANI layer is another important factor that can affect the biosensor performance. PANI, as a supporting material for enzyme immobilization, can enhance the active surface area contributing to an increase in enzyme loading. However, the excessive thickness of PANI can affect the diffusion of the analyte and electron transfer kinetics. The effect of the PANI thickness on the biosensor performance was examined by varying the number of electropolymerization scans at 0, 10, 20, 30, and 40 cycles. The results revealed that 20 scan cycles exhibited the highest sensitivity, as illustrated in Figure 3B. The sensitivity increased with an increase in the number of electropolymerization scans from 0 to 20 cycles. Then, a decrease in the sensitivity was observed when the number of scans increased from 20 to 40 cycles. This could possibly be because the excessive granules of the PANI layer lead to an increase in the background current; therefore, the current response decreases. The results were confirmed by SEM images showing the surface morphology of the PANI layers at different scan cycles (Supplementary Materials Figure S1). This indicated that the higher the number of scans, the higher the density of granules distributed on the electrode surface. Thus, the PANI with the number of electropolymerization scans at 20 cycles was chosen for further studies.

#### 3.2.3. Effect of PU Concentrations

To enhance the applicability of our developed sensor for use in human blood samples covering the sepsis range, we employed polyurethane (PU) as an outer membrane to extend the linearity of detection. The effect of PU concentration on the sensitivity and linearity was examined at 0% (non-PU), 1.0%, 2.0%, and 3.0% PU concentrations (*w*/*v*). Figure 3C illustrates the plots between the sensitivity and the linearity at the different PU concentrations. Non-PU cases exhibited the highest sensitivity but the linear range of detection was up to 1 mmol L^−1^, which did not cover the sepsis range. In contrast, the addition of the PU on the LOx/PANI/*m*-PD/SPAuE extended the linearity of detection by slowing the mass transport of the analyte. The results revealed that an increase in PU concentrations resulted in a decrease in sensitivity but an increase in linearity. This was consistent with a study reported earlier [28]. This finding could be explained that as the PU concentration increased, the PU layer became more viscous and the background current response increased, resulting in a significant reduction in its sensitivity. Regarding the developed lactate biosensors at the 1.0%, 2.0%, and 3.0% PU, they exhibited a wider linear range of lactate detection (0.2–2.0 mmol L^−1^, 0.2–5.0 mmol L^−1^, and 0.2–8.0 mmol L^−1^, respectively) as compared to that of the non-PU case (0.2–1.0 mmol L^−1^). This suggests that the addition of a PU layer can limit the diffusion of the lactate on the electrode surface. Also, the limitation of mass transfer can possibly affect the kinetics of the enzyme catalytic system, leading to a wider range of lactate detection. However, controlling the fabricated lactate biosensor at the 3.0% PU concentration posed challenges due to its high viscosity, leading to rapid solvent evaporation and difficulty in reproducing the process. Additionally, the response time of the lactate detection for each condition was also evaluated based on T_90_ (the time required for the current signal to reach 90% of the final value due to an increase in lactate). The response times at non-PU, 1.0%, 2.0%, and 3.0% PU concentrations were 2.30 ± 0.47 s, 3.50 ± 0.50 s, 5.0 ± 0.82 s, and 13.50 ± 1.50 s, respectively. Taking into account sensitivity, linear range, and response time, the 2.0% PU concentration was chosen as the optimal condition for further studies. This is because it exhibited a desirable sensitivity with a short response time (5.0 s), and a linearity covering the range of 0.20–5.0 mmol L^−1^, making it suitable for clinical applications, especially sepsis screening [31].

#### 3.2.4. Effect of Operational Potential for Lactate Determination

A series of constant potentials within the range of 0.50 to 0.80 V was systematically investigated and optimized based on sensitivity. The operational potential plays a critical role in the oxidation of H_2_O_2_, a by-product of the catalytic activity of lactate oxidase. The LOx acts as a catalyst of the lactate reaction, to oxidize lactate into pyruvate and H_2_O_2_. In the presence of oxygen, H_2_O_2_ is oxidized at the electrode surface by applying a constant potential and electrons are generated as given below in Equations (1) and (2) [12]:

(1) $$\text{L-lactate}+O_{2}\overset{\mathrm{LOx}}{\to }\;\mathrm{Pyruvate}+H_{2}O_{2}$$

(2) $$H_{2}O_{2}\overset{\;}{\to }O_{2}+2H^{+}+2e^{-}$$

Figure 3D illustrates that the highest sensitivity for the developed biosensor in lactate detection was achieved at 0.70 V. Consequently, a constant potential of 0.70 V was chosen for lactate determination for analytical performance studies.

### 3.3. Analytical Performances of the Optimized PU/LOx/PANI/m-PD/SPAuE

#### 3.3.1. Linearity and Detection Limit

**Figure 4:**
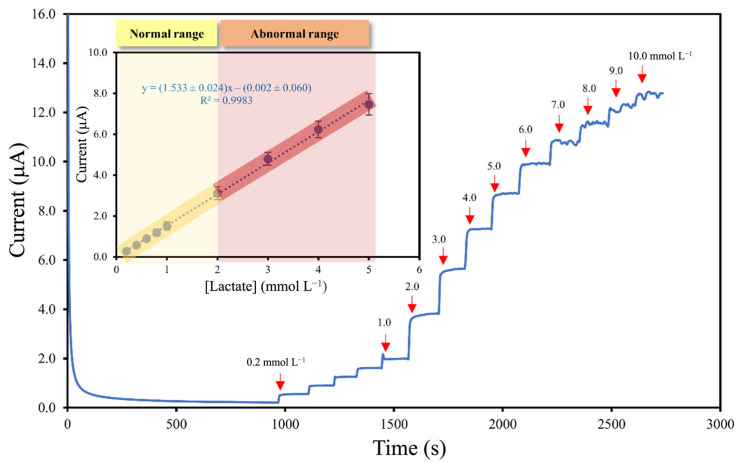
The current response of the PU/LOx/PANI/*m*-PD/SPAuE at different concentrations of lactate using amperometry with an inset representing the calibration plot of the developed biosensor for lactate detection covering normal range and abnormal range of lactate level (n = 3).

Under the optimal conditions, the performance of the developed lactate biosensor (PU/LOx/PANI/*m*-PD/SPAuE) was evaluated at different lactate concentrations in the range of 0.2–10 mmol L^−1^. The amperometric response obtained at a constant potential of +0.70 V is depicted in Figure 4. The result revealed that the current response exhibited a proportional increase with the concentration of lactate. As illustrated in the calibration plot inset, the biosensor exhibited a linear detection range from 0.20 to 5.0 mmol L^−1^ with a correlation coefficient of 0.9983. The sensitivity was 12.17 ± 0.02 µA mmol^−1^ L cm^−2^ with the limit of detection (LOD) of 7.9 µmol L^−1^ (3S/N) [32].

#### 3.3.2. Selectivity

**Figure 5:**
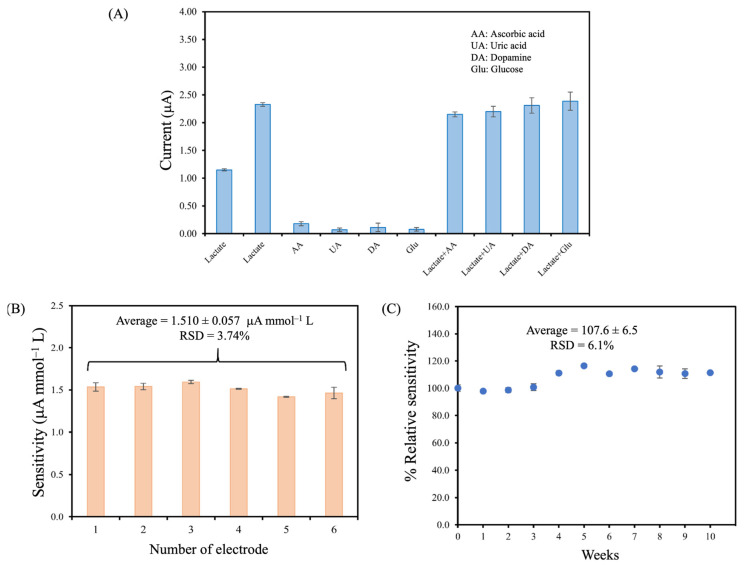
(**A**) Interference testing of PU/LOx/PANI/*m*-PD/SPAuE biosensor in 0.10 mol L^−1^ PBS containing different concentrations of the interfering substrates, including 1.0 mmol L^−1^, and 2.0 mmol L^−1^ lactate, 0.10 mmol L^−1^ AA, 0.10 mmol L^−1^ UA, 0.10 mmol L^−1^ DA, 5.0 mmol L^−1^ Glu, and also testing in the mixture solution of 2.0 mmol L^−1^ lactate with these interferents, respectively, (**B**) reproducibility of six lactate biosensors on the sensitivity at 0.20–5.0 mmol L^−1^ lactate using amperometry and, (**C**) the stability testing for the detection of 0.40–3.0 mmol L^−1^ lactate.

The selectivity of the developed lactate biosensor was evaluated by determining potential interfering species existing in blood plasma samples. These include ascorbic acid (AA), uric acid (UA), dopamine (DA), and glucose (Glu). In this study, the selectivity of the interfering species was determined at the high physiological concentration containing 0.10 mmol L^−1^ AA, 0.10 mmol L^−1^ UA, 0.10 mmol L^−1^ DA, and 5.0 mmol L^−1^ Glu. As shown in Figure 5A, the results revealed that there was no interfering effect on the developed lactate biosensor. This is possibly because *m*-PD as a perm-selective membrane is a protection layer, which helps limit access of both cations as well as anion molecules, and possible electrochemical species interference. Also, it allows H_2_O_2_, as a product of the lactate reaction, to pass through to the electrode surface. However, the developed lactate biosensor was sensitive to the analyte [18,26]. This indicates that the developed biosensor can be used for lactate determination.

#### 3.3.3. Reproducibility and Long-Term and Storage Stability

The reproducibility of the developed biosensor was evaluated by preparing six biosensors and measuring the current response at different lactate concentrations (0.20–5.0 mmol L^−1^). The sensitivities of the six developed lactate biosensors were 1.534 ± 0.050, 1.540 ± 0.038, 1.594 ± 0.019, 1.513 ± 0.005, 1.418 ± 0.007, and 1.463 ± 0.686 (Figure 5B). The average sensitivity was 1.510 ± 0.057 µA mmol^−1^ L, with a relative standard deviation (RSD) of 3.74%, indicating an acceptable reproducibility [33].

One of the developed sensors was assessed weekly to explore its long-term stability based on relative sensitivity (>90% of the initial testing is accepted value of AOAC guideline [34]). As seen in Figure 5C, the result showed that the relative sensitivity of the developed biosensor remained stable over 90% for the first four weeks. Subsequently, there was an approximately 10% increase in sensitivity from the initial value and this elevated sensitivity was sustained over the following six consecutive weeks (weeks 5–10). The initial increase in relative sensitivity during the first period could be attributed to the slight swelling of the PU membrane caused by its immersion in a PBS solution after each testing cycle. This resulted in the diffusion of the solution into the PU membrane, thereby increasing membrane permeability. However, a stable relative response was observed after the initial four weeks. The diffusion behavior can be described by the Fickian diffusion, where solution diffusion through the membrane is influenced by the thickness of the membrane [35,36]. The average relative sensitivity over the entire 10-week period was calculated to be 107.6 ± 6.5 with an RSD of 6.1%. These results indicated that the developed biosensor provided excellent long-term stability for over 10 weeks.

The storage stability was investigated by preparing nine lactate biosensors (PU/LOx/PANI/*m*-PD/SPAuE) at the same time and storing them in a sealed system at 4 °C in 0.10 mol L^−1^ PBS (pH 7.4) when not in use. The developed lactate biosensor was randomly examined one test at a time. Initially, the lactate measurements were conducted every 3–5 days and then every 10 days for two months. The storage stability was evaluated based on relative sensitivity. The results showed that the average relative sensitivity of 103.4 ± 4.0% with an RSD of 3.9% was obtained (Supplementary Materials Figure S2). Thus, the PU membrane layer as a protective membrane could prevent the enzyme from leaching and enhance the storage stability of the developed lactate biosensor. The results were consistent with previous studies [37,38], which reported that the characteristics of PU as the outer membrane characteristics could improve stability.

#### 3.3.4. Comparison with Other Sensors

The analytical performance of the PU/LOx/PANI/*m*-PD/SPAuE as a lactate biosensor was compared with other studies, as shown in Table 1. Our developed lactate sensor exhibited a wide linearity for lactate detection, distinguishing it from previously cited reports. While the linear range was not as extensive as some similar works [39], it was sufficient for early sepsis diagnosis via lactate detection. In terms of stability, this sensor outperformed other cited reports. In addition, the developed lactate sensor offers a fast response time (5.0 s) as compared with other studies [40] (30 s). This performance gives it a high potential for clinical lactate detection compared to the sensors evaluated in the cited works.

#### 3.3.5. Analysis of Human Blood Plasma Samples

**Figure 6:**
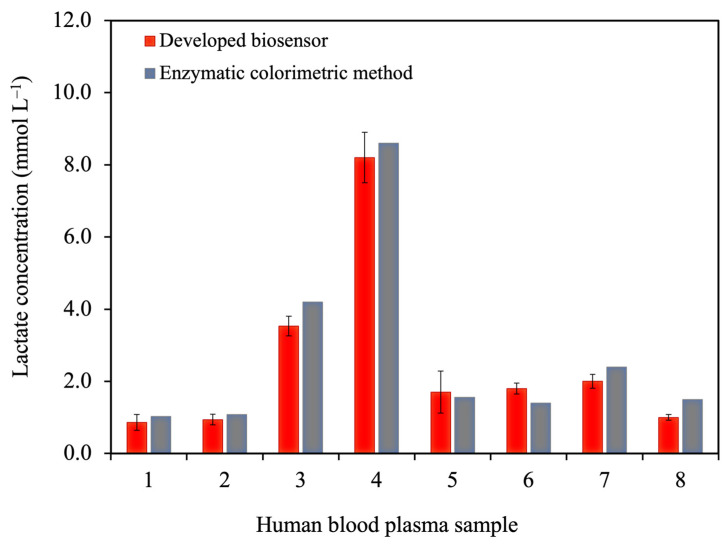
Comparison of the analytical results obtained from the developed biosensor and the enzymatic colorimetric method for lactate measurements in blood plasma samples.

In this study, the standard curve of the developed lactate biosensor exhibited a linear range of 0.20 to 5.0 mmol L^−1^; therefore, human blood plasma samples were diluted 10 times before testing. Initially, the matrix effect was evaluated by comparing the slope of the calibration curve of standard lactate solutions with that of the spike samples. The results showed that the sensitivity of the standard lactate solution and spike sample had no significant difference (*p* > 0.05, two-way ANOVA). Thus, the dilution at 10 times had no matrix effect. The lactate values of the diluted samples were then calculated from the calibration equation of the lactate standard curve. The lactate values of eight blood plasma samples obtained from the developed biosensor and the enzymatic colorimetric technique as a gold standard method in clinical use (from the Songklanagarind hospital) were in good agreement (*p* > 0.05), as illustrated in Figure 6. Notably, expanding the dataset in future studies is crucial for a comprehensive evaluation of false positive and false negative rates in our lactate detection method. An extended clinical study is in progress to yield valuable insights, enhancing the overall impact of our work.

The developed lactate biosensor was analyzed to verify the practicality using the recovery test based on the standard addition method. The recoveries were found to be in the range of 93.2–106.4% (Supplementary Materials Table S1), which were the accepted values according to the AOAC guidelines (80–110%) [33]. This indicates that the developed lactate biosensor exhibits high accuracy for lactate detection.

## 4. Conclusions

This study presents the successful fabrication of a lactate-based electrochemical biosensor, using a polymeric layer-by-layer method with *m*-PD, PANI, and PU modifications on a screen-printed gold electrode. The developed lactate biosensors exhibited excellent sensitivity, selectivity, reproducibility, long-term stability, and high accuracy of recovery in lactate detection. In addition, the developed biosensor could effectively detect the concentration of lactate in blood plasma samples compared to the enzymatic colorimetric method. With a wide linear response (0.2–5.0 mmol L^−1^) and a low limit of detection (7.9 µmol L^−1^), this biosensor holds promise for early sepsis diagnosis. It stands as an efficient and reliable tool for the development of portable lactate point-of-care testing, facilitating on-site early sepsis diagnosis.

## Figures and Tables

**Table 1:** The comparison of performance of lactate biosensors based on lactate oxidase (LOx).

Electrode Materials	Linear Range (mmol L^−1^)	Detection Limit (µmol L^−1^)	Sensitivity (µA·mmol^−1^ L cm^−2^)	Stability (Days)	Samples	Ref.
LOx/MWCNTs/CuNPs/PANI/PEG	0.0010–2.5	0.25	NR	140	Blood plasma	[41]
LOx-Cu-MOF/CS/Pt/SPCE	0.00075–1.0 4.0–50.0	0.75	116.26 1.64	50	Sweat, saliva, wine	[42]
LOx/TiO_2_ sol gel-Gr/Ni foam	0.050–10.0	19	NR	8	Commercial rabbit serum	[39]
LOx-PVA-SbQ/*m*-PD/Pt disk	0.005–1.0	5	81.6	14	Human blood serum	[40]
LOx/Pt/PANI/MXene/SPCE	0.005–5.0	5	0.62	30	Milk samples	[24]
LOx-CNDs/SPAuE	0.003–0.50	0.9	39.52	NR	Human blood serum	[43]
PU/LOx/PANI/*m*-PD/SPAuE	0.20–5.0	7.9	12.17	>70	Human blood plasma	This work

MWCNTs: multiwalled carbon nanotubes; CuNPs: copper nanoparticles; PEG: pencil graphite electrode; Cu-MOF: copper metallic framework; CS: chitosan; SPCE: screen-printed carbon electrode; PVA-SbQ: photopolymer-containing styrylpyridine groups; CNDs: carbon nanodots; NR: not reported.

## Data Availability

Data are contained within the article and supplementary materials.

## Supplementary Materials

The following supporting information can be downloaded at: https://www.mdpi.com/article/10.3390/polym16040473/s1, Figure S1: SEM images of PANI electropolymerization at different numbers of scans: (A) 10 cycles, (B) 20 cycles, (C) 30 cycles, and (D) 40 cycles, (E) cyclic voltammograms of PANI electropolymerization at four different number of scans; Figure S2: The storage stability testing for the detection of 0.40–3.0 mmol L^−1^ lactate in 0.1 mmol L^−1^ PBS (pH 7.4) using amperometry; Table S1: The recovery study was performed using the standard addition method. Figure S3: Comparison of the calibration curves between the standard lactate solution and the spiked lactate in three blood samples.
