# Supplementary material for: Disposable Polyaniline/m-Phenylenediamine-Based Electrochemical Lactate Biosensor for Early Sepsis Diagnosis
Source: Polymers (Basel). 2024 Feb 8;16(4):473. doi: 10.3390/polym16040473 (PMC10892424; doi:10.3390/polym16040473)
Supplement: Supplementary file 1 [file polymers-16-00473-s001.zip › polymers-2828182-supplementary.pdf]

Supplementary data

Disposable Polyaniline/*m*-Phenylenediamine-Based Electrochemical Lactate Biosensor for Early Sepsis Diagnosis

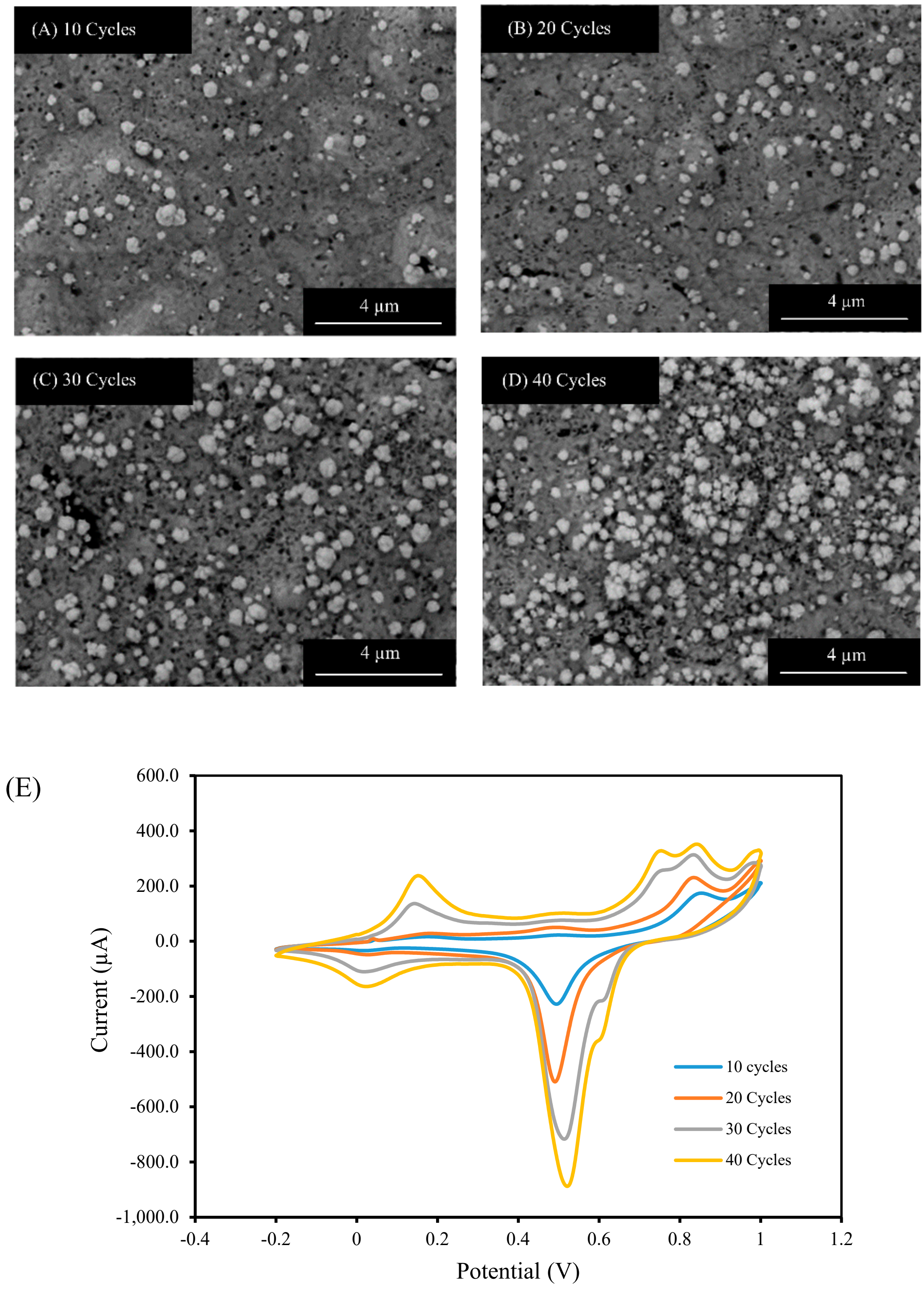

Figure S1. SEM images of PANI electropolymerization at different number of scans: (A) 10 cycles (B) 20 cycles (C) 30 cycles, and (D) 40 cycles. (E) Cyclic voltammograms of PANI electropolymerization at four different number of scans

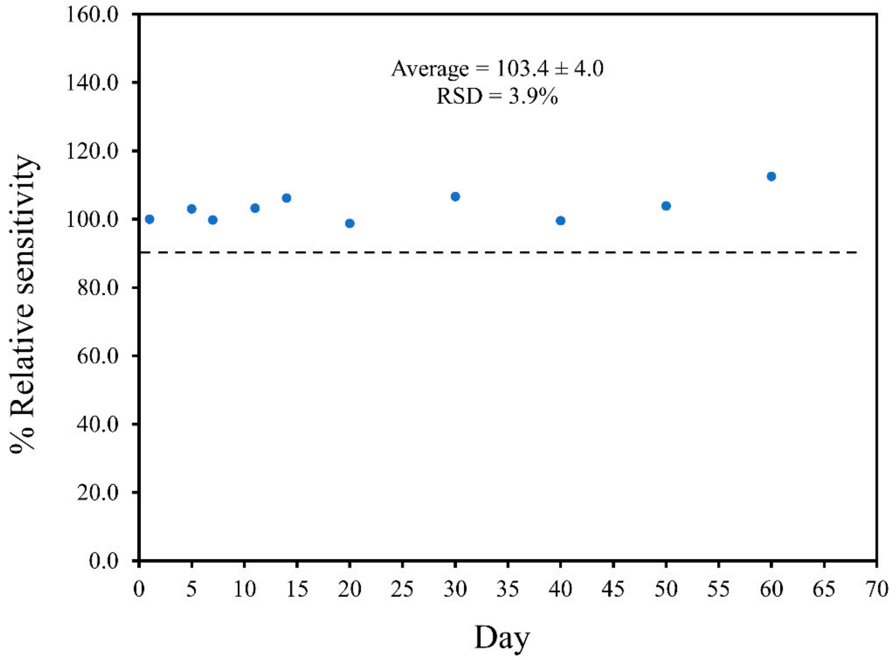

Figure S2. The storage stability testing for the detection of 0.40 - 3.0 mmol L^{-1} lactate in 0.1 mmol L^{-1} PBS pH 7.4 using amperometry.

Table S1. The recovery study was performed using the standard addition method.

Spiked concentration (mM)	Sample 1		Sample 2		Sample 3	
	Measurement Concentration (mM) ± S.D (n=3)	Recovery (%) ± S.D (n=3)	Measurement Concentration (mM) ± S.D (n=3)	Recovery (%) ± S.D (n=3)	Measurement Concentration (mM) ± S.D (n=3)	Recovery (%) ± S.D (n=3)
0.40	0.41±0.06	103.6±3.5	0.38±0.07	93.9±1.49	0.42±0.16	104.5±3.68
0.60	0.56±0.05	93.2±3.7	0.6±0.09	99.7±0.03	0.57±0.18	95.4±4.71
1.0	1.06±0.14	106.3±4.6	0.97±0.08	97.0±2.77	0.99±0.11	99.2±2.62
2.0	2.01±0.09	100.4±4.6	2.13±0.10	106.4±1.37	2.03±0.07	101.7±3.48
3.0	2.99±0.29	99.5±6.4	2.93±0.25	97.6±0.89	2.98±0.06	99.4±2.55

Sample 1:

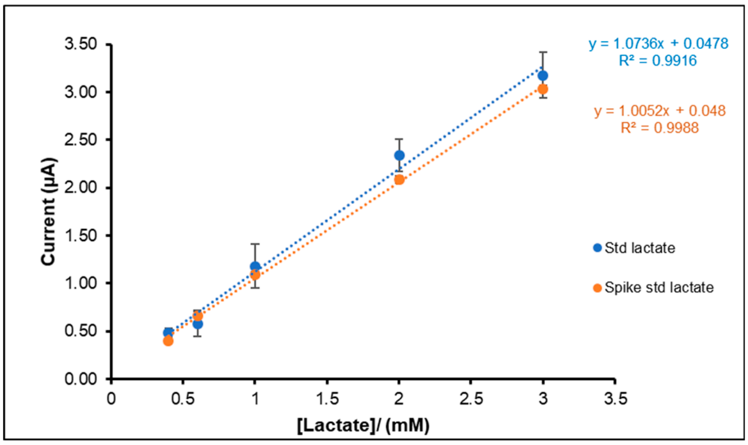

Sample 2:

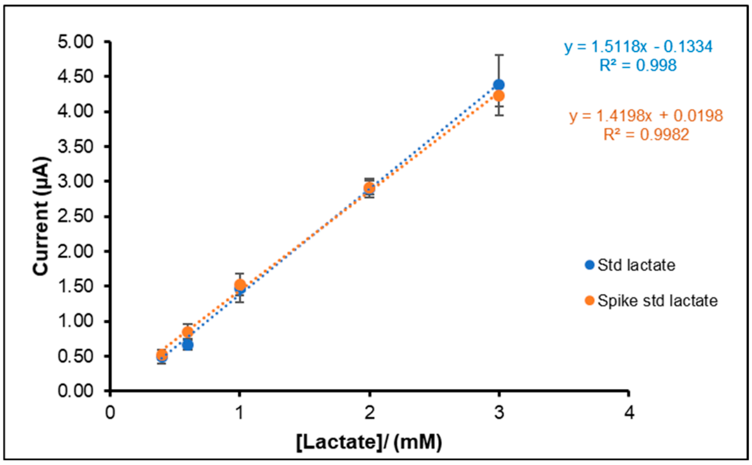

Sample 3:

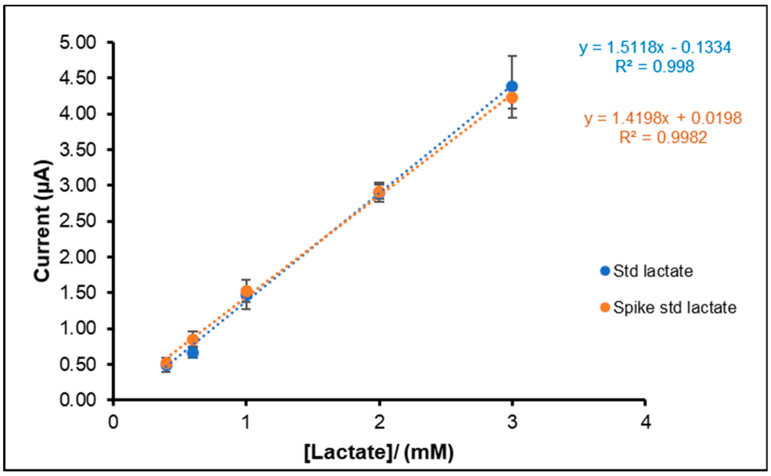

Figure S3: Comparison of the calibration curves between the standard lactate solution and the spiked lactate in three blood samples.
